# The need for disruptive innovation in acute kidney injury

**DOI:** 10.1007/s10157-020-01920-w

**Published:** 2020-06-24

**Authors:** Kent Doi

**Affiliations:** Department of Emergency and Critical Care Medicine, The University of Tokyo, 7-3-1 Hongo, Bunkyo, Tokyo 113-8655 Japan

**Keywords:** Biomarker, Animal model, Critical care nephrology, Organ crosstalk

## Abstract

Acute kidney injury (AKI) is a threatening medical condition associated with poor outcomes at different settings. The development of standardized diagnostic criteria and new biomarkers addressed significant clinical impacts of AKI and the need for an early AKI detection, respectively. There have been some breakthroughs in understanding the pathogenesis of AKI through basic research; however, treatments against AKI aside from renal replacement therapy (RRT) have not shown adequate successful results. Biomarkers that could identify good responders to certain treatment are expected to facilitate translation of basic research findings. Most patients with severe AKI treated with RRT died due to multiple-organ failure, not renal dysfunction. Hence, it is essential to identify other organ dysfunctions induced by AKI as organ crosstalk. Also, a multidisciplinary approach of critical care nephrology is needed to evaluate a complex organ crosstalk in AKI. For disruptive innovation for AKI, we further explore these new aspects of AKI, which previously were considered outside the scope of nephrology.

## Introduction

Acute kidney injury (AKI) is characterized by an acute decline in renal function. Since the concept of AKI was suggested by addressing the importance of early detection and early intervention around 2004–2005 [1, 2], many clinical studies have provided evidences that AKI is significantly associated with poor outcomes in various clinical situations [3]. This has increased awareness on AKI in the area of nephrology and other areas such as critical care medicine and cardiology. Basic research detected pathophysiological pathways that contributed to the development of AKI and subsequent progression of kidney injury using sophisticated techniques such as genetic engineering [4], intravital microscopy [5], and comprehensive analysis of transcriptomics [6] and metabolomics [7]. In AKI diagnosis, several new biomarkers have been clinically introduced and earlier detection of AKI has become possible in the last decades. However, no significant translation from the bench to the bedside has been attained in therapeutics against AKI. Thus, desired results of AKI research that would provide significant improvements on the outcomes of AKI patients are yet to be achieved.

## Diagnosis

### Serum creatinine and urine output

The Kidney Disease Improving Global Outcomes (KDIGO) criteria were published in 2012 and is currently the international gold standard of AKI diagnosis [8]. The KDIGO criteria were established by integrating two previous AKI diagnosis criteria: the Risk, Injury, Failure, Loss of kidney function, and End-stage kidney disease (RIFLE) [9] and the Acute Kidney Injury Network (AKIN) [10] criteria. Recently, the Japanese clinical practice guideline for AKI was developed [11]. In this guideline, the KDIGO criteria were evaluated in comparison with the RIFLE and the AKIN criteria. In eleven observational studies, the KDIGO criteria were found to be more accurate than, or as accurate as, the RIFLE and AKIN criteria in predicting the in-hospital mortality of patients. Of note, these observations were mostly noted from the data of inpatients (hospital-acquired AKI). Community-acquired AKI was found to be more severe AKI than hospital-acquired AKI and showed better outcomes such as mortality despite having similar risk factors [12, 13].

**Fig. 1:**
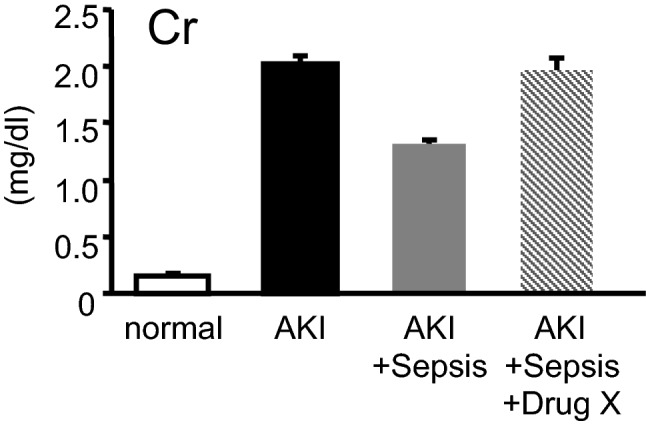
Paradoxical increases of serum creatinine by sepsis treatment. Creatinine production in muscle is suppressed by sepsis and creatinine secretion into urine is reduced by septic AKI. If drug X improves sepsis and septic AKI, both creatinine production and secretion will be increased. These responses hamper the accurate evaluation of drug X against septic AKI. Cr, serum creatinine

The KDIGO criteria defined AKI as an increase in the serum creatinine and a reduction in the urine output. It is well known that an increase in serum creatinine delays glomerular filtration rate (GFR) reduction for 24–48 h [14]. To overcome this, the kinetic estimated GFR calculation was suggested [15]. Several studies evaluated its clinical significance in critically ill patients [16–18]. However, serum creatinine may not be elevated in a reduced GFR since creatinine production is suppressed under sepsis condition [19]. Sepsis is a leading cause of AKI in ICUs [20]. Hence, serum creatinine and serum creatinine-based methods are neither early nor sensitive markers of AKI especially in ICUs (Fig. 1).

The KDIGO criteria determines AKI severity by serum creatinine elevation and urine output. The Japanese AKI guideline [19] indicated that urine output along with serum creatinine significantly improved the survival outcome predictions in ICU patients. It suggested that the urine output should be included in AKI severity evaluation whenever possible. However, no study on outpatients or general ward patients has been conducted to evaluate the urine output criteria. Although urine output can be measured hourly in ICUs, it is not feasible to obtain urine output in other clinical settings such as the emergency department and general ward.

Two common clinical markers of serum creatinine and urine output, which are used by AKI diagnosis with the KDIGO criteria, have several serious disadvantages as described above. This promoted to develop better diagnostic tools for AKI and several new AKI biomarkers were investigated for the last decade.

### Biomarker

Although AKI is a syndrome associated with a broad spectrum of diseases and a variety of underlying etiologies, two distinct phenotypes need to be assessed in clinical settings: transient (“pre-renal”) AKI and persistent (“renal”) AKI. Transient AKI can be categorized as a fluid-responsive AKI as correction of renal perfusion by fluid resuscitation will rapidly improve renal function in transient AKI patients. In several different clinical conditions, the prognosis of transient AKI was reportedly better than persistent AKI [21–25]. Persistent AKI is generally diagnosed with more severe AKI stage, i.e., higher serum creatinine and lower urine output (oliguria or anuria) compared with transient AKI. Distinguishing transient over persistent AKI is crucial for clinical management and for enrollment of AKI clinical trials because including transient AKI patients in the trials will interfere with detecting potential drug effects. However, determining transient over persistent AKI requires a fluid challenge or certain observation time. Fluid challenge may increase the risk of pulmonary edema and mechanical ventilation may be required when it is complicated with heart failure. A longer observation time will decrease the chance of response to new AKI treatment in clinical trials.

Especially in ICUs, the common underlying etiology of persistent AKI is acute tubular injury. Detecting tubular epithelial injury helps in distinguishing persistent from transient AKI. The fractional excretion of sodium (FeNa) and urea (FeUN) are widely used; however, their effectiveness is inadequate especially in sepsis [26, 27]. Furthermore, GFR reduction and subsequent serum creatinine elevation will be observed after tubular injury in AKI. For early detection and differentiation of persistent AKI, monitoring the tubular epithelial cell injury is expected to be a better strategy than monitoring the serum creatinine and urine output in AKI diagnosis based on the KDIGO criteria. Under these considerations, new AKI biomarkers have been developed (Table 1). Post-cardiac surgery AKI has been evaluated for the early detection of AKI using new biomarkers, because the incidence of renal insult is obvious (i.e., cardiopulmonary bypass) as well as a relatively high prevalence of AKI after the surgery (30–40%). Neutrophil gelatinase-associated lipocalin (NGAL) and L-type fatty acid-binding protein (L-FABP) were approved by the Japanese health insurance system. Both reportedly detected AKI earlier than serum creatinine elevation in adult and pediatric post-cardiac surgery AKI [28–35]. Animal experiments demonstrated that expressions of these biomarkers in the kidney were significantly increased in the persistent AKI model (ischemia reperfusion and cisplatin injection) than in the transient AKI model (dehydration) [36, 37]. Urinary NGAL can help in differentiating persistent AKI from transient AKI in a cohort from the emergency department [38]. In studies on inpatients, several biomarkers including NGAL and L-FABP showed statistically significant difference between pre-renal AKI and renal AKI, but some overlaps were observed between these two groups [36, 39, 40]. Similar overlaps were observed between non-AKI and AKI especially in adult ICU cohorts, possibly because of background heterogeneity and unclear onset of renal insult [41–43]. These findings suggest that measuring one biomarker cannot perfectly predict AKI development or other outcomes.

**Table 1:** Newly developed biomarkers

Biomarker	Characteristics		Sample type	Clinical trial setting					Clinical use
				ICU	Cardiac surgery	Kidney transplant	Contrast media	ER	
NGAL	25-kDa polypeptide	1) Produced by neutrophils, liver, spleen and kidney (tubular cell) 2) Inhibiting bacterial growth, scavenging iron, inducing epithelial cell growth 3) Filtered by glomerulus and taken up by the proximal tubule through megalin	Blood and urine	+	+	+	+	+	Approved in Europe, Japan and Canada
IL-18	18.3-kDa cytokine	1) Produced by immune cells and active epithelial cells 2) Caspase-1 cleaves pro-IL-18 into the active IL-18 molecule	Urine	+	+	+	?	?	Not approved for clinical use
L-FABP	14-kDa cytosolic protein	1) Filtered by glomerulus and taken up by the proximal tubule 2) Acts as a carrier protein and transports free fatty acids to mitochondria and peroxisomes	Urine	+	+	?	?	+	Approved in Japan
KIM-1	39-kDa type-1 transmembrane protein	1) Expressed in proximal tubule cells and is thought to promote apoptotic and necrotic cell clearance 2) By injury, upregulated and shed into the urine and extracellular space	Urine	?	+	–	?	+	Not approved for clinical use
[TIMP-2]x [IGFBP7]	TIMP-2, 21-kDa; IGFBP7, 29-kDa	1) Inducers of G1 cell cycle arrest 2) Expressed in epithelial cells and act in an autocrine and paracrine manner to arrest cell cycle in AKI	Urine	+	+	?	?	?	Approved in United States and Europe

**Fig. 2:**
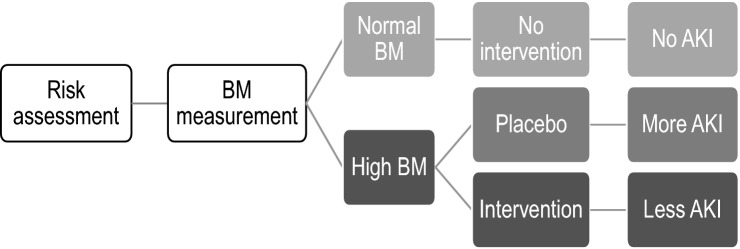
Use of biomarkers for enrollment in clinical trials. *BM* biomarker

In cancer screening, five consecutive phases of biomarker development were proposed [44]. The final phase (Phase 5) describes whether biomarker measurement reduced the burden of disease on the population. Even if AKI was detected early through the biomarker, an overall benefit needs to be identified with the impact of measurement on improving the outcomes of AKI. Since no specific drug that can prevent or treat AKI is clinically available, including biomarker measurement in AKI clinical trials may be a possible solution (Fig. 2). After clinical risk factors are evaluated, patients are then screened for eligibility by measuring biomarkers. Prevalence of renal tubular injury (i.e., high biomarker value) will increase the proportion of target patients at enrollment and decrease the sample size and trial cost. If drug A will be demonstrated to be effective in patients with high biomarker B (responder), both drug A and biomarker B will simultaneously be considered. Several clinical trials on renal replacement therapy (RRT) and bundle approach against AKI have recently used biomarkers as basis for patient enrollment [45, 46]. The PrevAKI study aimed to evaluate the effectiveness of implementing the KDIGO guideline bundle in preventing post-cardiac surgery AKI in high-risk patients characterized by the urinary insulin-like growth factor-binding protein 7 (IGFBP7) and tissue inhibitor of metalloproteinases-2 (TIMP-2) [46]. A significant reduction in AKI occurrence was reported in the intervention group. It should be noted that 495 of 882 screened patients were excluded in this study due to low biomarker values.

Early detection of AKI seemed to be the main purpose for new AKI biomarker development; however, other purposes such as predicting fluid responsiveness (differential diagnosis of renal versus prerenal AKI) and identifying responders to certain treatments need to be emphasized for clinical applications.

## Treatment

### Barrier to translation

As abovementioned, no clinically available drug can prevent or treat AKI. A number of potential effective treatments in basic research with animal models failed to be translated for clinical application. It has been pointed out that the failure to translate results from basic research to clinical practice is due to complex and heterogeneous disease characteristics of AKI, inappropriate designs of clinical trials (e.g., delayed drug administration, canceled therapeutic benefit by adverse events, and inadequate sample size), and irrelevant animal models that insufficiently mimic human AKI [47].

The need for clinically relevant animal models in the preclinical study has been addressed [48, 49]. Sepsis and AKI are highly complicated syndromes, having several different pathological mechanisms; hence, the replication of septic AKI in animal models is difficult. Exogenous lipopolysaccharides (LPS) administration causes immune responses in animals that are similar to human sepsis and septic shock. An LPS injection induced a rapid and significant increase of the blood inflammatory cytokines TNF-α and IL-1β, and treatments with neutralizing antibodies against these mediators significantly reduced mortality and organ injuries in LPS-injected mice. However, these treatments failed to show any protection from human sepsis [50, 51].

Cecal ligation and puncture (CLP) is another animal model showing a ligation distal to the ileocecal valve and a needle puncture of the ligated cecum that causes fecal material to leak into the peritoneum, resulting in poly-microbial bacteremia and sepsis [52]. Supportive treatment as with fluids and antibiotics is often given to mimic human sepsis. CLP-induced sepsis models show a cytokine profile similar to that of human sepsis [53], and an anti-TNF-α treatment fails to alleviate sepsis in CLP models similar to that in the case of human sepsis [54, 55]. However, CLP does not cause AKI in young C57/BL6 mice, prompting the use of either aged C57/BL6 or outbred CD-1 mice for the CLP-mediated induction of septic AKI [56, 57]. Chronic kidney disease (CKD) is a strong risk factor for AKI development in various clinical settings [58, 59]. To address the impact of preexisting renal dysfunction on sepsis and sepsis-induced AKI, a two-hit animal model of CKD-sepsis (acute-on-chronic renal failure) has been developed [60, 61]. In the CKD-sepsis model, some potential therapeutic targets of vascular endothelial growth factor (VEGF) and high-mobility group box 1 (HMGB1) have been identified.

Animal models should mimic human AKI in as many aspects as possible, including the pace and severity of disease, renal hemodynamic changes, histological findings, and immunological responses. The use of bigger animals such as sheep and pigs may be applicable to mimic the human ICU environment, especially in the later phase of drug development. More fundamentally, to develop AKI models of higher clinical relevance, other factors that should be mimicked must be considered. Due to a very limited number of renal biopsies occurring in human AKI, actual mechanisms of disease and drug targets for human AKI could be overlooked. Of note, the Kidney Precision Medicine Project (KPMP), funded by the National Institute of Diabetes and Digestive and Kidney Diseases (NIDDK), is trying to obtain kidney biopsy samples from AKI patients to better characterize human AKI [62].

Basic research is undoubtedly necessary to develop clinically effective treatments against AKI. One-way translation of from bench-to-clinical approach seemed not to be effective so far. Bedside-to-bench-to-bedside approach will enable to improve animal models in basic research and promote drug development in the clinical.

### Renal replacement therapy and blood purification

When potentially fatal changes in the body fluid, electrolyte, and acid–base balance occur, renal replacement therapy (RRT) is an effective treatment for severe AKI. Because of the remarkable progress in RRT, hemodynamically unstable patients in the ICU may be treated with continuous RRT (CRRT) or sustained low-efficiency dialysis (SLED) [63, 64]. Data obtained from the Japanese Diagnosis Procedure Combination database in 2011 showed that approximately 80% of adult dialysis-requiring AKI (AKI-D) patients in ICUs initially received CRRT [65]. Unfortunately, the mortality rate of AKI-D patients was unacceptably high (40–50%), although it gradually decreased in the last decades [66, 67]. Similar finding of gradual mortality reduction was reported in all the AKI population, whose mortality was approximately 20–30% [68]. Several clinical studies were conducted to determine the optimal RRT dose, modality, and timing for AKI-D; however, accumulated evidences did not provide a clear conclusion and recent AKI guidelines do not have a recommended standard RRT therapeutic strategy [8, 11].

Direct hemoperfusion using polymyxin B-immobilized fiber column (PMX-DHP) has been used for septic shock. Because AKI is a major complication of septic shock, PMX-DHP was expected to improve septic AKI. Several observational studies using the Japanese diagnosis procedure combination database showed controversial results [69, 70]. However, a recent meta-analysis that utilized low-risk-of-bias clinical trials showed that PMX-DHP had no effect on survival [71]. Other adsorption filters such as AN69ST and Cytosorb^Ⓡ^ were evaluated in the clinical settings [72–74], even though their quality of evidence is not high. Further evaluations on these new filters are necessary.

Although RRT is considerably effective on severe AKI complicated with life-threating conditions, AKI-D patients still show unacceptably high mortality rate. Related blood purification therapy failed to demonstrate clinical utility for improvement of the outcomes. These suggest that breakthroughs are necessary for these treatments using extracorporeal circulation techniques.

## Beyond the kidney

### Critical care nephrology

**Fig. 3:**
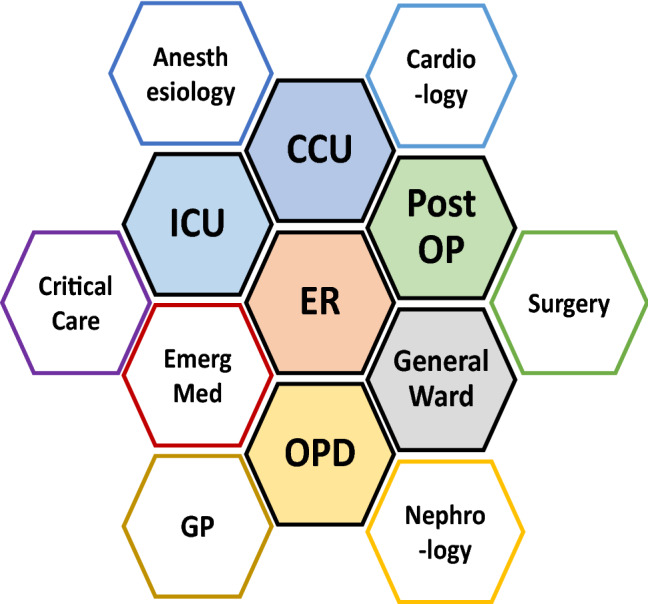
Critical care nephrology involves different specialties and locations in the management of AKI patient. *GP* general practitioner, *OPD* outpatient department

In 1998, Ronco and Bellomo published a paper on their personal opinion on the need for “critical care nephrology,” which was suggested by these authors as a multidisciplinary approach to AKI [75]. They pointed out that effective clinical management for AKI requires a collaboration between intensivists and nephrologists. Moreover, for this multidisciplinary team development, they suggested a structure of training and patient care for critical care nephrology. An excellent training program on critical care nephrology is available at Alberta University, Edmonton, Canada [76]. The Acute Dialysis Quality Initiative (ADQI) Group, which suggested the RIFLE criteria [9], consisted of both intensivists and nephrologists [8]. Also, the KDIGO guideline for AKI was suggested by these two different specialists. However, the care for AKI patients in ICUs varied largely across institutions, and the role of nephrologists in critical care was recently determined by the American Society of Nephrology Acute Kidney Injury Advisory Group [77]. It should be noted that not only nephrologists and intensivists but also other specialists such as cardiologists, anesthesiologists, general surgeons, hospitalists, and emergency physicians need to be involved in critical care nephrology (Fig. 3).

In Japan, some nephrologists worked for acute dialysis in ICUs and some intensivists developed CRRT techniques for their clinical settings [78]. The 2016 Japanese AKI guideline was a result of collaboration of five different societies: the Japanese Society of Nephrology, the Japanese Society of Intensive Care Medicine, the Japanese Society of Dialysis Therapy, the Japanese Society of Blood Purification in Critical Care, and the Japanese Society of Pediatric Nephrology [11]. However, there is a concern as regards the career development as a critical care nephrologist in Japan. In the current system developed by the Japanese Medical Specialty Board, it is necessary to complete two independent programs or internal medicine and emergency medicine/anesthesiology to be a double-board nephrologist and intensivist.

Critical care nephrology is a new specialty that promotes a multidisciplinary approach to AKI. There is no doubt that critical care nephrology is important and necessary to improve the outcomes of AKI patients. However, this specialty has not been widely accepted since Ronco and Bellomo suggested it in 1998. Further exploration will strengthen the role of critical care nephrology.

### Organ crosstalk

In ICU patients, AKI does not occur alone but most often develops as a one-organ injury in multiple-organ failure. Although AKI in ICU patients is associated with a high mortality, other factors other than loss of kidney function seem to contribute to poor outcomes because non-AKI-D patients still had a remarkably higher mortality than the end-stage renal disease (ESRD) patients [79]. As previously described, uremic conditions can be controlled through advanced technique of RRT even in unstable patients. In spite of the prevalence of technical advances, poor outcomes of AKI-D have been reported worldwide [66, 67, 80, 81]. These data indicate the following possibilities: (1) still insufficient replacement of renal function by current RRT, (2) considerably higher impact of other organ injuries on mortality than AKI, and (3) unrecognized mechanisms such as amplification of distant organ injury by AKI (organ crosstalk).

Organ crosstalk between the kidney and the heart is clinically recognized as a clinical entity called cardiorenal syndrome (CRS) [82]. CRS includes both renal failure secondary to cardiac dysfunction and heart failure secondary to renal dysfunction. Type 1 CRS includes AKI occurred in acute heart failure (low cardiac output syndrome). Type 3 CRS is characterized by an acute reduction of renal function that leads to acute cardiac injury and/or dysfunction. Retention of uremic solutes and/or volume overload may contribute to heart injury in type 3 CRS.

Organ crosstalk induced by AKI has been analyzed in the field of basic research. In the last decade, a number of experimental studies using animal models have identified possible pathways that connect the kidney with other organs such as the lung, heart, spleen, and gut (Table 2) [83]. For the kidney–lung interaction, inflammatory pathways such as IL-6 [84], neutrophil activation [85], TNFR1-mediated apoptosis [86], and toll-like receptor 4–high-mobility group protein B1 (HMGB1) [87] were identified. Metabolomic changes are intensively examined in the kidney of AKI patients [7]; however, similar changes were observed in the heart of AKI patients in the context of kidney–heart interaction [88, 89]. As no specific drug is clinically effective against AKI, targeting these organ-crosstalk pathways is expected to lead to novel therapeutics against AKI and decrease the high mortality of AKI-related multiple-organ failure. Before this translational step, the significance of each organ-crosstalk pathway in human study should be confirmed.

**Table 2:** Mechanisms of organ crosstalk in AKI

Lung	Immune cell (neutrophil, T cell, and macrophage) infiltration and NETs formation
	Systemic IL-6 elevation and pulmonary CXCL1 expression
	TLR4 activation by HMGB1
Heart	Cellular apoptosis by cardiac TNF-α expression
	Mitochondrial fragmentation and apoptosis by dynamin-related protein 1 upregulation
Spleen	Supporting protective effect of TLR9 inhibitor chloroquine
	Secretion of HMGB1 and IL-10
	Transient reservoir of mobilized transient endothelial progenitor cells
	Cholinergic anti-inflammatory pathway activation by exposure of the kidney to ultrasound waves
Brain	Increased chemokine expression of keratinocyte-derived chemoattractant and granulocyte colony-stimulating factor in cortex and hippocampus
	TLR4 upregulation in hippocampus and striatum
	Anti-inflammatory reaction by vagal nerve stimulation
Liver	Leukocyte infiltration, increased oxidative stress, and hepatocyte apoptosis
	Altered cytochrome P450 3A enzyme activity
Gut	Production of protective short-chain fatty acid for renal injury by microbiota
	Paneth cell activation (IL-17A production)

## Perspective

New concepts of AKI and standardized diagnosis criteria helped us to determine a considerable impact of AKI on clinical outcomes and the need for early detection and early intervention. New biomarkers are a more useful tool in monitoring renal injury in AKI compared with serum creatinine and urine output. However, a better tool for therapeutics against AKI remains unknown. How can we make a breakthrough in this situation? The term of “disruptive innovation” was defined and first analyzed in 1995 by the American scholar Clayton M. Christensen [90]. A disruptive innovation is defined as an innovation that creates a new market and value network that will disrupt an already existing market and replace an existing product. How will a disruptive innovation occur in AKI? Highly advanced RRT technique is ultimately useful for life-threating conditions in severe AKI, and it seems that the “AKI therapeutic market” is satisfied with this high-end product. Unfortunately, RRT cannot effectively treat approximately 50% of AKI-D patients. In basic research, several studies investigated on the possible protective effects in kidney injury, but they did not pay much attention on other organ injuries in AKI. Exploring targets aside from the kidney may provide a disruptive innovation for AKI treatment.

Extracellular histones and neutrophil extracellular traps (NETs) are one of the potential targets in other organ injuries in AKI. Extracellular histones induce cytotoxicity triggering the inflammatory cascade via toll-like receptors. They are released from neutrophils during the NETs formation. In a mouse intestinal ischemia–reperfusion model, we observed extracellular histone accumulation and NETs formation in the liver rather than intestine [91]. Extracellular histones derived from the intestinal tract were considered to transported to the liver via the portal system. In mouse renal ischemia–reperfusion injury model, elevated extracellular histones in blood and NETs formation in the lung was observed [92]. Human recombinant thrombomodulin (rTM) is clinically applied as a therapeutic agent for disseminated intravascular coagulation. Human rTM reportedly trap extracellular histones in vitro. Although no renal protection by rTM was observed in renal ischemia–reperfusion model, significant improvement of lung injury together with NTEs accumulation induced by AKI was observed in rTM-treated animals [93]. Taken together, extracellular histones and NETs formation seem to be responsible for AKI-induced lung injury and these pathways may be a good drug target for suppressing organ crosstalk in AKI.

In conclusion, AKI is one of the most serious diseases that show unacceptably high mortality and no specific drug can sufficiently prevent and treat AKI in the clinical. We need to be aware that AKI is not a self-limited kidney disease but a syndrome with wide disease spectrum mostly complicated with systemically ill patients. On the other hand, many basic researches especially focusing on organ crosstalk in AKI have found new mechanisms. Hopefully, a multidisciplinary approach of critical care nephrology and remote organ injury evaluation in AKI will lead to new directions for future AKI research.
